# Stereotactic radiosurgery for brain metastases: multistate and competing risk models of progression and survival from a single-centre 11-year experience

**DOI:** 10.1186/s13014-025-02779-5

**Published:** 2026-02-05

**Authors:** Dharsshini Reveendran, Vassili Crispi, Sam Fairclough, Paul Hatfield, Ryan K. Mathew

**Affiliations:** 1 Department of Neurosurgery, Leeds General Infirmary, Leeds Teaching Hospitals NHS Trust, Leeds, UK; 2 School of Medicine, University of Leeds, Leeds, UK; 3 Adult Neurology, Leeds General Infirmary, Leeds Teaching Hospitals NHS Trust, Leeds, UK; 4 Leeds Cancer Centre, St James’ University Hospital, Leeds Teaching Hospitals NHS Trust, Leeds, UK

**Keywords:** Stereotactic radiosurgery, SRS, Gamma Knife, Brain metastases

## Abstract

**Background:**

The diagnosis of brain metastases has risen due to advances in systemic treatments prolonging survival and the use of MRI. Whilst surgical resection remains a valuable mean of acutely alleviating raised intracranial pressure, reducing seizure risk, and preventing progression of neurological deficit, stereotactic radiosurgery (SRS) delivers radiotherapy to precise target volumes, including the treatment of a larger number of metastases with high rates of local control, whilst avoiding the neurological complications of WBRT or the need for invasive resection. The aim of this study was to evaluate the 11-year experience of SRS at the Leeds Gamma Knife Centre, examining patient outcomes, and the association of patient-dependent factors and intracranial disease burden to survival.

**Methods:**

A retrospective cohort study of 1,031 patients (2,836 metastases) treated with SRS at the Leeds Gamma Knife Centre between 2010 and 2020. Data included primary tumour, Karnofsky performance status, intracranial disease burden, and prior/subsequent treatment. Follow-up was at least 30 months. Kaplan-Meier analysis was used for 10-year survival curves. Multistate and competing risks models assessed progression/death predictors.

**Results:**

Lung (50.4%), breast (15.4%) and melanoma (9.9%) were the most common primary tumour. 364 (35.3%) patients had recurrence, and 875 (84.5%) died by the end of follow-up. Survival outcomes were worse in men compared to women in both lung (*p* = 0.003) and other (*p* = 0.02) metastases. Breast cancer patients spent the most time in the no progression state (*rmean* = 1.56 years) whereas gastrointestinal primary spent the least (*rmean* = 0.99 years). Each additional metastatic lesion at the time of treatment increased the risk of progression by 9.2% (*p* < 0.001), but risk of death was not influenced by the number of lesions (*p* = 0.30); conversely, total tumour volume increased the risk of death by 2.2% for each 1cm^3^ increase. Post-SRS new metastatic disease increased the risk of death by 31% (*p* = 0.02), whilst local recurrence did not (*p* = 0.26).

**Conclusion:**

These findings add to the accepted prognostic factors for brain metastases, further informing clinical consultations with patients, but further research is required to address unanswered questions regarding the role of SRS in relation to other treatments.

## Introduction

The diagnosis of brain metastases has risen due to advances in systemic treatments prolonging survival and the use of MRI. An increasing number are asymptomatic, with metastases identified on screening scans [1]. With a prevalence of 20–40% in patients with metastatic cancer, brain metastases are a significant cause of mortality and morbidity, predominantly arising from lung, breast, melanoma, kidney, and gastrointestinal tract cancers [1].

The management of brain metastases is dependent upon the number, site, and size of the lesions, the patient’s baseline performance status (PS), previous and prospective treatment, and the stability and prognosis of their extracranial disease [2]. Reducing tumour volume is the primary aim in the management of brain metastatic disease: a reduction of brain metastatic burden has been strongly linked to improved neurocognitive function, neurological function, and overall survival. Treatment should be delivered in the safest possible way, with as few side effects as possible [3].

Surgical resection remains a valuable means of acutely alleviating raised intracranial pressure, whilst also reducing seizure risk, preventing progression of neurological deficit and reducing duration of steroid use [4]. Whilst patients with a solitary metastasis benefit from longer survival and decreased recurrence following resection, the benefit in patients with multiple metastases is minimal [5]. Here, whole brain radiotherapy (WBRT) has been the primary treatment for brain metastases, despite its negative impact on cognition and quality of life [6]. Whilst surgery followed by WBRT has been reported to significantly improve survival and functional independence compared to WBRT alone, WBRT alone offers minimal additional benefit compared to supportive care [6]. In contrast, stereotactic radiosurgery (SRS) delivers radiotherapy to precise target volumes, including the treatment of a larger number of metastases with high rates of local control, whilst avoiding the neurological complications of WBRT or the need for invasive resection [4, 7–9]. Hence, the American Society of Radiation Oncology (ASTRO) 2022 guidelines recommend SRS over WBRT as initial treatment for patients who present with less than 4 brain metastases and have an Eastern Cooperative Oncology Group (ECOG) performance status of 0–2 [10].

SRS may also play a wider role in patients’ treatment. Recent studies found that SRS achieves better locoregional control, e.g. progression-free survival, than with or without additional WBRT, and patients undergoing SRS following surgical resection had prolonged median survival compared to those receiving WBRT after resection [11–13]. Furthermore, SRS in patients with single or multiple brain metastases lengthens survival compared to surgical resection [14, 15]. Given the recent advances in SRS companion technologies, such as improved image guidance and collimation capabilities, the precision of SRS has increased its clinical application in otherwise surgically inaccessible sites, and SRS has been associated with prolonged quality of life for up to 12 months when achieving locoregional control and stabilisation of cerebral symptoms [16, 17].

This study aimed to evaluate the 11-year experience of SRS at the Leeds Gamma Knife Centre, examining patient outcomes, and the association of patient-dependent factors and intracranial disease burden to survival with a long prospective follow-up using multistate models and a competing risks analysis.

## Method

This is a single-centre, retrospective, observational cohort study.

### Patient selection

An initial 1,113 patients with brain metastases who underwent Gamma Knife SRS at the Leeds Cancer Centre between January 2010 and December 2020 were identified. Patients were referred if they had a Karnofsky performance status (KPS) ≥ 70 and a prognosis of greater than 6 months as defined by the clinical team treating their primary tumour. The number of brain metastases, the total volume of the metastases, the patient’s baseline performance status, and their options for systemic treatment were also taken into consideration when selecting patients for SRS.

### Radiosurgery technique

On the day of treatment, most patients were fitted with a Leksell stereotactic G frame under local anaesthetic. Lorazepam (0.5-1 mg) was offered as necessary. Since 2015, the Leeds Cancer Centre has used Gamma Knife ICON©, which also accommodates immobilisation using a relocatable mask as necessary. Generally, this has only been used to fractionate the treatment of larger metastases or for rare patients where pin placement was not feasible after prior surgery.

T1-weighted gadolinium-enhanced MRI brain scans were performed and analysed by a clinical oncology consultant and radiologist to guide contouring and dose selection. Planning was performed using *Gammaplan*^®^ planning software. Metastatic targets were treated to doses ranging from 18 to 24 Gy depending on size and position, with lower doses used for larger lesions or those close to organs at risk such as the optic tracts. When fractionation was used it was either given as 27 Gy in 3 fractions or 30 Gy in 5 fractions. Doses were prescribed to an appropriate isodose line to achieve optimal conformity and to achieve a steep dose drop off outside the treated volume. Prophylactic anti-epileptics were not administered. Patients who were already on steroid therapy for symptomatic relief were given a weaning regimen after treatment.

### Patient data collection

Data on patient demographics, KPS at the time of referral, primary tumour histology, previous treatment, number and volume (in mm^3^) of brain metastases were recorded. Patients were followed up with regular MRI of the brain every 3 months in the first 12 months post-SRS, every 4 months in the second year post-SRS and every 6 months in years 3, 4 and 5. Patients’ follow-up and survival status were recorded as of June 2022, and the minimum follow up length was 2.5 years. During the follow up, the followings were recorded: intracranial disease progression following SRS, subsequent SRS or neurosurgical intervention; treatment toxicity as recorded in clinic or subsequent admissions to hospital; survival status (alive/dead); and cause of death. Follow-up MRI of the brain and the radiology reports were analysed to assess for the presence of local recurrence or new distant metastases: to discriminate between radionecrosis and local recurrence, subsequent MRI were also scrutinised to evaluate the evolution of the SRS treated region of interest over time. The date of the MRI which first identified any local or new distant disease was recorded as the date of recurrence. Due to variations in referral to SRS and pre-SRS record keeping, it was not possible to assess how many patients received WBRT prior to SRS. However, post-SRS WBRT data was collected. The study data was anonymised at collection.

### Statistical analysis

Statistical analysis was undertaken using the statistical software package ‘*R*’ (available via https://www.r-project.org/, version 4.3.1). All analysis was performed using base ‘*R*’ and the ‘*survival*’ package. Survival curves and p values were generated in ‘R’ using the ‘*survfit*’ and ‘*survdiff*’ functions respectively. Survival was defined as time in months from date of first Gamma Knife treatment to date of death. Statistical significance was identified as *p* < 0.05.

### Model and variable selection

Kaplan-Meier analysis was performed in R, to provide a visual depiction of 10-year survival based on primary tumour type alone. For each primary tumour type a p-value was calculated comparing male and female subjects.

**Fig. 1:**
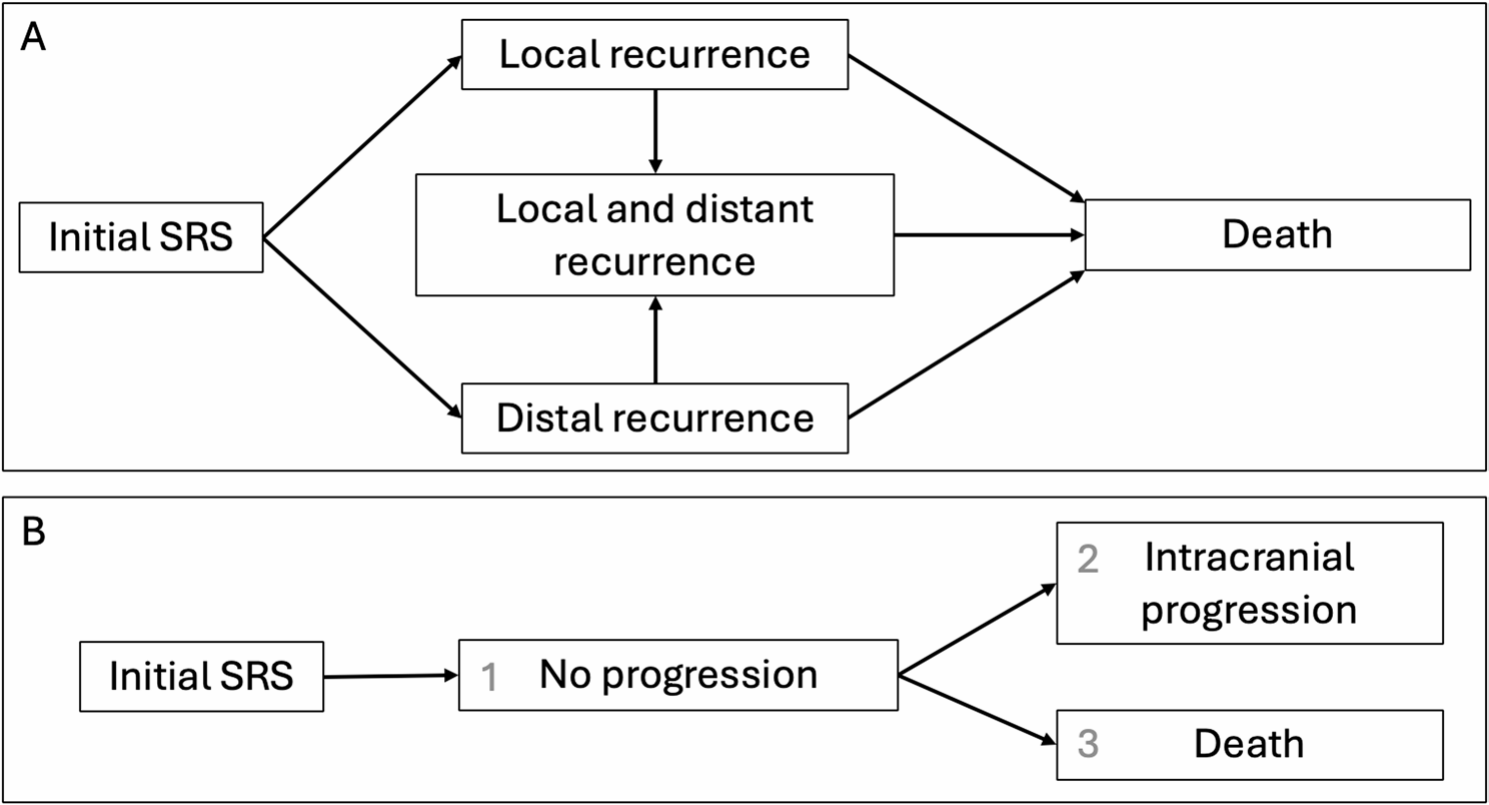
**A**: Multistate model flow diagram depicting time-dependent movement between states to either local or distal recurrence towards a final absorbing state of ‘death’. **B**: Competing risks flow diagram depicting movement between competing states, preventing individuals from progressing from state 1 into states 2 and 3 simultaneously. SRS, stereotactic radiosurgery

A multistate model was created using the ‘tmerge’ command in which death was the absorbing state and the data arranged in a counting style. Time dependent covariates were also created to assess whether an individual subject had experienced either (a) recurrence at the original SRS treated site (local recurrence) or (b) recurrence at a new site distal to that originally treated (distal recurrence) (Fig. 1A). A Cox-proportional hazards analysis was undertaken to better quantify whether moving into either of the recurrence states influenced the risk of death. As other variables in the dataset were likely to influence overall survival, both patient age at treatment and primary tumour type were included in the final model using stratification via the ‘*strata*’ function. Calculation of Schoenfeld residuals demonstrated that these fixed variables left un-stratified would break proportional hazard assumptions. Total tumour volume (cm^3^) was also included in this model and did not violate proportional hazard assumptions.

A competing risks model was also designed to assess the effect of several variables on two competing events, (a) progression of any sort (local or distal) and (b) death. Again the ‘tmerge’ function and counting style arrangement of the data were employed with 3 states of ‘No progression’, ‘Intracranial progression’ and ‘Death’ possible (Fig. 1B). Code was included to prevent individuals from progressing into states 2 and 3 simultaneously, however, on manual checking of the data this did not appear to occur regardless. Checking transitions between states, there were no duplicates or ‘backwards’ moves that would not be logical. Total tumour volume and number of lesions at the time of treatment were assessed for significance on the two events of interest. Two stratified co-variables were added to improve the model; ‘primary tumour diagnosis’ and ‘age at SRS’ as both could increase the risk of death from other causes. Schoenfeld residuals were within acceptable limits for all non-stratified variables for each of the possible transitions.

## Results

### Patient demographics

A total of 1,031 patients who received treatment to a total of 2,836 brain metastases were included in the final study and multistate and competing risk models. 82 patients were excluded from the study due to missing or incomplete data. The median (IQR) age of the patient cohort at the time of initial SRS was 64 (55–71) years. There were slightly more female patients than male (57.3% vs. 42.7%) included in the study. A breakdown of their demographics by primary tumour type can be seen in Table 1.

**Table 1:** Patient demographics by primary tumour type

	Gastrointestinal	Renal	Breast	Melanoma	Lung	Others*
**Number (%)**	85 (8.3)	88 (8.5)	159 (15.4)	102 (9.9)	520 (50.4)	77 (7.5)
**Age (years**,** median (IQR))**	66 (55–73)	65 (58–72)	55 (48–64)	62 (52–71)	67 (59–73)	63 (50–69)
**Sex (%)**						
Male	57 (67.1)	62 (70.5)	4 (2.5)	60 (58.8)	226 (43.5)	31 (40.3)
Female	28 (32.9)	26 (29.5)	155 (97.5)	42 (41.2)	294 (56.5)	46 (59.7)
**KPS (%)**						
60	0	0	1 (0.7)	0	5 (1.1)	0
70	8 (11)	7 (10.6)	13 (9.4)	5 (5.8)	63 (14.2)	8 (11.9)
80	5 (6.8)	12 (18.2)	12 (8.6)	6 (7)	63 (14.2)	3 (4.5)
90	37 (50.7)	30 (45.4)	54 (38.8)	36 (41.9)	165 (37.2)	26 (38.8)
100	23 (31.5)	17 (25.8)	59 (42.4)	39 (45.3)	147 (33.2)	30 (44.8)
**Total Number of lesion(s)**						
1–3	74 (87.1)	73 (83)	123 (77.4)	60 (58.8)	401 (77.1)	59 (76.6)
4–9	10 (11.8)	11 (12.5)	32 (20.1)	32 (31.4)	99 (19)	15 (19.5)
> 9	1 (1.2)	4 (4.5)	4 (2.5)	10 (9.8)	20 (3.8)	3 (3.9)
**Total tumour volume (cm** ^**3**^ **)**						
< 5	20 (23.5)	34 (38.6)	47 (29.6)	37 (36.3)	228 (43.8)	31 (40.3)
≥ 5–10	28 (32.9)	37 (42)	50 (31.4)	30 (29.4)	158 (30.4)	22 (28.6)
≥ 10–15	10 (11.8)	11 (12.5)	37 (23.3)	18 (17.6)	70 (13.5)	11 (14.3)
≥ 15–20	16 (18.8)	5 (5.7)	12 (7.5)	8 (7.8)	34 (6.5)	7 (9.1)
≥ 20–25	6 (7.1)	0	10 (6.3)	6 (5.9)	23 (4.4)	2 (2.6)
≥ 25–30	3 (3.5)	1 (1.1)	2 (1.3)	0	4 (0.8)	0
> 30	2 (2.4)	0	1 (0.6)	3 (2.9)	3 (0.6)	4 (5.2)
**Prior surgery**	6 (7.1)	4 (4.5)	19 (11.9)	6 (5.9)	16 (3.1)	7 (9.1)
**Post-SRS WBRT**	5 (6.7)	3 (4.1)	27 (18.4)	7 (7.4)	20 (4.3)	3 (4.6)
**Complications (%)**						
None	32 (59.3)	24 (38.1)	44 (34.9)	33 (40.7)	179 (48.5)	20 (37)
Dizziness	3 (5.6)	0	13 (10.3)	6 (7.4)	25 (6.8)	2 (3.7)
Seizure	0	2 (3.2)	6 (4.8)	6 (7.4)	21 (5.7)	2 (3.7)
Fatigue	18 (33.3)	33 (52.4)	69 (54.8)	37 (45.7)	142 (38.5)	29 (53.7)
Headache	1 (1.9)	2 (3.2)	2 (1.6)	3 (3.7)	11 (3)	2 (3.7)
Alopecia	2 (3.7)	0	1 (0.8)	1 (1.2)	3 (0.8)	0
**Intracranial disease progression**						
New metastases (%)	16 (18.8)	24 (27.3)	66 (41.5)	41 (40.2)	157 (30.2)	28 (36.4)
Local recurrence (%)	3 (3.5)	3 (3.4)	13 (8.2)	3 (2.9)	25 (4.8)	3 (3.9)
Months to new metastases (median (IQR))	11 (3.5-25.25)	6 (5.75-13)	6 (3.75–10.5)	5 (3–8)	6 (3–12)	9 (5.75–17.75)
Repeat SRS (%)	6 (8.1)	14 (19.4)	30 (20.7)	21 (22.3)	103 (22)	15 (22.4)
Months to repeat SRS (median (IQR))	12.5 (4.5-25.75)	7 (6.75–13.25)	8 (4.75–13.25)	6 (4–11)	7 (3–15)	9.5 (7.25–20.5)
**Mortality**						
Death due to intracranial disease progression (%)	11 (19)	10 (15.4)	43 (36.8)	23 (35.4)	71 (19.9)	16 (29.1)
Death due to extracranial disease progression (%)	47 (81)	55 (84.6)	74 (63.2)	42 (64.6)	285 (80.1)	39 (70.9)
**Overall survival (months (median (IQR))**	7 (3–12)	8 (2.75-18)	14.5 (6–27)	7 (3–17)	8 (3–17)	10 (3–22)

KPS, Karnofsky Performance Status; IQR, interquartile range; SRS, stereotactic radiosurgery; WBRT, whole brain radiotherapy. Others*: Bladder cancer, cervical cancer, head, and neck cancers

**Fig. 2:**
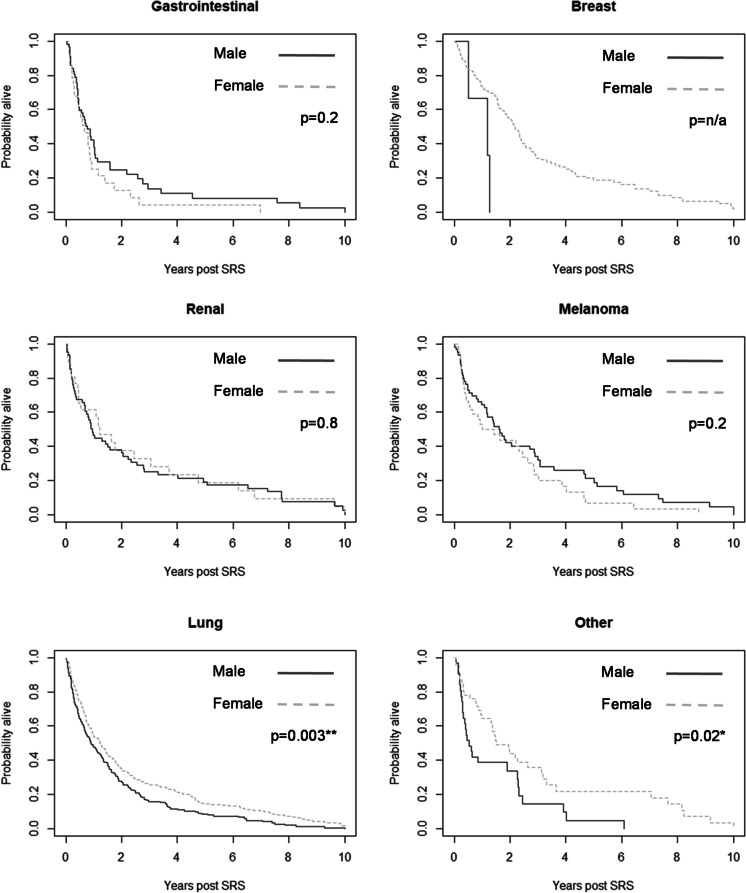
Kaplan-Meier plots showing 10-year overall survival by primary tumour type and sex. Chi square test performed comparing survival between sexes in each primary type are represented by p-values. SRS, stereotactic radiosurgery

Minimum follow-up period was 30 months. During follow-up, 364 of 1031 individuals developed new intracranial recurrence and 875 died. Amongst patients who were alive at study completion, the longest follow-up was 12 years and 4 months. Survival over 10 years was plotted on a Kaplan-Meier survival curve for each of the primary tumour types by sex (Fig. 2).

### Multistate model

A multistate model was used to estimate how the risk of death was influenced by either local recurrence at the SRS treated site or new ‘distal’ disease away from the original treatment zone. Developing distal metastatic disease increased the risk of death by 31% (HR 1.31, CI[1.04–1.64], *p* = 0.02). Conversely, local recurrence at the previous SRS site was not associated with an increased risk of death (*p* = 0.26). Each 1cm^3^ increase in the total tumour volume at the time of SRS was associated with a 1.5% increase in the risk of death (HR 1.015 CI[0.3%-2.8%], *p* = 0.01).

### Competing risks model

Of 1031 patients included in the competing risks model, 47 were censored without either event (progression or death) occurring. A total of 364 progressed with new intracranial disease with 875 (256 of whom had already progressed intracranially) dying at some point during follow up. The risk of moving into state 2 (intracranial progression) from state 1 (no progression) was not influenced by total tumour volume (*p* = 0.94). However, breast cancer patients spent the most time in the no progression state (*rmean* = 1.56 years) whereas gastrointestinal primary spent the least (*rmean* = 0.99 years).

The number of metastatic lesions at the time of treatment was associated with an increased risk of intracranial progression (*p* < 0.001). For each additional metastatic lesion, an increase in the risk of progression of 9.2% was seen. Conversely, the risk of death was not influenced by the number of lesions (*p* = 0.30). Total tumour volume increased the risk of death by 2.2% for each 1cm3 increase (*p* = 0.002).

## Discussion

Brain metastases are estimated to affect 30% of all cancer patients, most frequently occurring in patients who have lung, breast, colorectal, skin (melanoma), or renal tumours [18, 19]. Given the increased use of surveillance imaging, an ageing population and improved prognosis and treatment for many patients with extracranial tumours, the incidence of brain metastases is increasing [1]. In a previous preliminary study of the first 58 patients treated with SRS at our institution, we demonstrated that the total volume of metastatic disease was a better predictor of outcome than the number of metastases treated. However, two main limitations of the previous study were the small sample size and the short median follow-up of 55 weeks [2]. The aim of the current study was to update the results of the previous study with a significantly larger cohort of patients (1,031 patients) and a prolonged median follow-up.

The incidence of brain metastases based on primary tumour types in our study echoes the findings of the Surveillance Epidemiology and End Results (SEER) program, revealing that lung cancer and melanoma exhibit the highest propensity for intracranial involvement [20]. However, several studies have reported that over 50% or more of patients with metastatic HER2 + or triple-negative breast cancer will experience brain metastases along their clinical trajectory [21–23]. Due to the increased incidence of brain metastases in these subgroups of tumour sites, the National Comprehensive Cancer Network (NCCN) advises brain imaging via magnetic resonance imaging (MRI) at the time of oncologic diagnosis for specific malignancies and disease stages, including small cell lung cancer, non-small cell lung cancer, melanoma, testicular cancer, and angiosarcoma [20].

The least common primary tumour site in our cohort was gastrointestinal (85 patients, 8.3%). This is higher than the average incidence of brain metastases in patients with gastric/oesophageal cancers (1.7–3.9%) and colorectal cancers (2.1%) [24, 25]. However, our analysis found that patients with primary gastrointestinal tumours spent the least time in the no progression state (rmean = 0.99 years) compared to patients with a diagnosis of breast cancer who spent the most time in the no progression state (rmean = 1.56 years). This finding may be explained by the late diagnosis of brain metastases in patients with gastrointestinal cancers due to the lack of primary system-specific symptoms and the late diagnosis for disseminated metastatic disease, which in turn delays the initiation of treatment [24].

Brain metastases are often a late presentation in advanced extra-cranial tumours. Firstly, the impact of screening and improved systemic therapies, such as in breast cancer and melanoma, have influenced the improvement in overall survival of patients, who are more likely to experience more advance disease recurrence [26]. In addition, patients with brain metastases are likely to present after prior palliative chemotherapy for extracranial metastasis disease, which indicates that these patients have already exhausted effective treatment options for their primary tumour by the time brain metastases occur [24]. Several studies have demonstrated the impact of primary histology on overall survival once brain metastases have been identified [27–29]. However, there is an existing debate on the implications of number of metastases, intracranial disease volume and the role of SRS in this context .

During follow-up, 364 of 1031 patients (35.3%) who received SRS for brain metastases developed new intracranial disease, in agreement with other studies (10% to 50%) [30, 31]. In our study, new metastatic disease increased the risk of death by 30.1% (HR 1.31, CI[1.04–1.64], *p* = 0.02); conversely, local recurrence at the previous SRS site was not associated with an increased risk of death (*p* = 0.26). Whilst clinical focus is placed on diagnosis of recurrence, which is challenging since it is difficult to distinguish between true disease progression and radionecrosis on follow-up MRI scans, these findings place priority on early identification of new intracranial metastatic sites over local recurrence, in keeping with other published evidence [32, 33].

In this study, the greatest predictor of overall survival in patients with brain metastases remains the total intracranial tumour volume: an increased risk of death by 2.2% for each 1 cm^3^ increase (*p* = 0.002). This is directly linked to overall survival but not progression, which has also been observed in other populations [3, 27]. In contrast, similar to other published studies, our study found that the number of intracranial lesions is linked to the likelihood of future progression but does not influence overall survival [28, 34]. Similarly, Hirshman et al. reported that the sensitivity and specificity of predicting 1-year survival were significantly increased when largest intracranial tumour volume (LITV) in the Scored Index for Radiosurgery (SIR) scale was substituted with cumulative intracranial volume (CITV) [35]. Since pressure effects and related neurological dysfunction will increase as the overall volume of the brain metastases increases, this factor may potentially be significant. Increased tumour volume burden would result in a decline in performance status and increase the risk of neurological death.

Of note, this study observed a statistically significant difference in overall survival between men and women in lung and other (bladder, head and neck, and germ cell) primary tumours. This is reflective of survival outcomes favouring women over men in lung cancer [36]. However, the reason for these findings is not fully understood. Whilst non-statistically significant, similar overall survival differences are observed in renal and melanoma metastases in our cohort. Whilst our findings and the literature do not fully explain these findings, they are likely to reflect a broader disease-specific difference, yet to be investigated.

Lastly, targeted therapies and immune checkpoint inhibitors have strengthened extra- and intracranial control in several malignancies, enabling more personalised treatment [18, 37]. Whilst optimal management of intracranial metastases is influence by several factors, such as performance status, primary histology and control of the primary disease, further consideration might be given to treatment of high intracranial disease burden, e.g. number of metastases greater than 4. Although there is not enough data to demonstrate that WBRT is a better option than SRS alone for patients with multiple intracranial metastases, SRS may become a more prominent clinical consideration in this patient group [38]. Therefore, this may shift rationale away from WBRT for palliation towards SRS for preservation of cognitive function with minimal complications as demonstrated by our findings and improving survival outcomes [39].

### Strengths and limitations

Whilst demonstrating a predictive link with intracranial disease burden and survival, strengths and limitations are recognised.

Firstly, this study evaluates a large patient population with a large cumulative number of brain metastases at a single tertiary centre in the UK. Whilst these findings present a single-centre experience of SRS, our 11-year experience of treating brain metastases supported by extensive clinical data may be generalisable to other neuro-oncology centres. Furthermore, these findings are strengthened by being presented alongside a prolonged observational follow-up, therefore describing the natural history of disease progression, which will be informative to clinicians and patients alike.

Second, building upon existing literature, this study sought to investigate and evaluate predictive factors to overall survival in patients deemed suitable for SRS. Given the international lack of consensus on patient selection and large clinical variation in practice, this adds to the growing body of evidence. Whilst advances in systemic therapies, such as immunotherapy or targeted therapy, have altered the oncological landscape, this analysis was beyond the scope of this study. Furthermore, despite having been performed at a single institution, the findings demonstrated significant survival predictors of overall survival and progression-free survival with a dedicated competing risks analysis.

Lastly, the scope of this study was narrow, and the study cannot address topics of current interest in brain metastases, such as the role and potential toxicity of immunotherapy in the context of SRS, a comparison between pre- and post-operative SRS, fractionation, or the role of WBRT. In fact, only data on post-SRS WBRT could be collected. Ultimately, prospective observational or interventional studies are better suited to investigate some of these unanswered questions in this patient population, which are beyond the scope of this study.

## Conclusion

Management of brain metastases poses a major challenge; in this context, SRS may provide an effective therapeutic option. This study of our 11-year experience of SRS at the Leeds Cancer Centre has presented an observational analysis of disease progression and a multistate model and a competing risks analysis of patient-dependent factors and intracranial disease burden to survival. The findings highlighted worse overall survival in men, a higher risk of death associated with new distal intracranial metastatic deposits following SRS, and the association between risk of death and the total treated volume. In particular, our analysis demonstrated the longer progression-free survival amongst breast cancer patients and the association between the risk of progression to new or recurrent disease with the initial number of treated metastatic lesions. These findings add to the accepted prognostic factors for intracranial metastases, further informing clinical consultations with patients to provide individualised treatment, but further research is required to address unanswered questions regarding the role of SRS in relation to other treatments.

## Data Availability

No datasets were generated or analysed during the current study.
